# Deep learning for opportunistic, end-to-end automated assessment of epicardial adipose tissue in pre-interventional, ECG-gated spiral computed tomography

**DOI:** 10.1186/s13244-024-01875-6

**Published:** 2024-12-19

**Authors:** Maike Theis, Laura Garajová, Babak Salam, Sebastian Nowak, Wolfgang Block, Ulrike I. Attenberger, Daniel Kütting, Julian A. Luetkens, Alois M. Sprinkart

**Affiliations:** 1 Department of Diagnostic and Interventional Radiology, University Hospital Bonn, Bonn, Germany; 2 Department of Radiotherapy and Radiation Oncology, University Hospital Bonn, Bonn, Germany; 3 Department of Neuroradiology, University Hospital Bonn, Bonn, Germany

**Keywords:** Epicardial adipose tissue, Transcatheter aortic valve replacement, Tomography (X-ray computed), Deep learning

## Abstract

**Objectives:**

Recently, epicardial adipose tissue (EAT) assessed by CT was identified as an independent mortality predictor in patients with various cardiac diseases. Our goal was to develop a deep learning pipeline for robust automatic EAT assessment in CT.

**Methods:**

Contrast-enhanced ECG-gated cardiac and thoraco-abdominal spiral CT imaging from 1502 patients undergoing transcatheter aortic valve replacement (TAVR) was included. Slice selection at aortic valve (AV)-level and EAT segmentation were performed manually as ground truth. For slice extraction, two approaches were compared: A regression model with a 2D convolutional neural network (CNN) and a 3D CNN utilizing reinforcement learning (RL). Performance evaluation was based on mean absolute z-deviation to the manually selected AV-level (Δz). For tissue segmentation, a 2D U-Net was trained on single-slice images at AV-level and compared to the open-source body and organ analysis (BOA) framework using Dice score. Superior methods were selected for end-to-end evaluation, where mean absolute difference (MAD) of EAT area and tissue density were compared. 95% confidence intervals (CI) were assessed for all metrics.

**Results:**

Slice extraction using RL was slightly more precise (Δz: RL 1.8 mm (95% CI: [1.6, 2.0]), 2D CNN 2.0 mm (95% CI: [1.8, 2.3])). For EAT segmentation at AV-level, the 2D U-Net outperformed BOA significantly (Dice score: 2D U-Net 91.3% (95% CI: [90.7, 91.8]), BOA 85.6% (95% CI: [84.7, 86.5])). The end-to-end evaluation revealed high agreement between automatic and manual measurements of EAT (MAD area: 1.1 cm^2^ (95% CI: [1.0, 1.3]), MAD density: 2.2 Hounsfield units (95% CI: [2.0, 2.5])).

**Conclusions:**

We propose a method for robust automatic EAT assessment in spiral CT scans enabling opportunistic evaluation in clinical routine.

**Critical relevance statement:**

Since inflammatory changes in epicardial adipose tissue (EAT) are associated with an increased risk of cardiac diseases, automated evaluation can serve as a basis for developing automated cardiac risk assessment tools, which are essential for efficient, large-scale assessment in opportunistic settings.

**Key Points:**

- Deep learning methods for automatic assessment of epicardial adipose tissue (EAT) have great potential.
- A 2-step approach with slice extraction and tissue segmentation enables robust automated evaluation of EAT.
- End-to-end automation enables large-scale research on the value of EAT for outcome analysis.

**Graphical Abstract:**

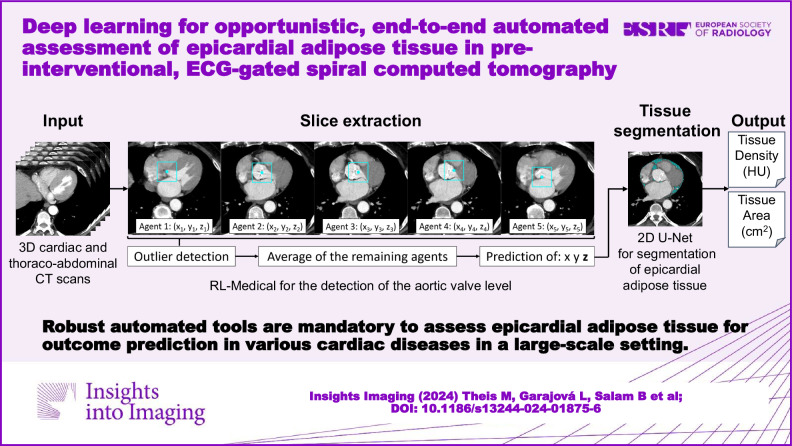

## Introduction

The volume and attenuation of cardiac adipose tissue (CAT) measured by CT has recently been shown to be a predictive marker for the outcome of various cardiac diseases [1–9]. For the assessment of CAT, a clear separation is made between epicardial adipose tissue (EAT), defined as adipose tissue between the myocardium and the pericardium, and pericardial adipose tissue (PAT), which refers to adipose tissue outside the pericardium. In the past, it has been shown that the EAT volume in particular is associated with coronary artery disease, myocardial ischemia, myocardial infarction or major adverse cardiac events [1–5]. Increased EAT volume was also found to be an independent predictor of all-cause mortality in patients undergoing transcatheter aortic valve replacement (TAVR) [6, 7]. In these studies, EAT volume was assessed manually or semi-manually by contouring the pericardium and applying appropriate thresholds or fully automatically using non-open, non-free commercial software [2–4].

The use of deep learning (DL) methods for the automation of quantitative image analyses enables an opportunistic large-scale assessment, which has already proven its worth in body composition analysis [10–15]. Also, the automation of volumetric EAT segmentation was investigated in several studies [16–18]. Recently, Haubold et al presented the open-source body and organ analysis (BOA) pipeline as an extension of the “TotalSegmentator” framework, which enables automated body composition analysis, including the assessment of EAT and PAT [19–21].

Previous research has shown a high correlation between 3D and 2D measurements at specific anatomical positions [9, 22, 23]. Therefore, one way to reduce time-consuming annotation effort in case of manual analyses is to evaluate EAT on a single slice instead of assessing EAT in the whole volume. Salam et al demonstrated the value of single-slice CAT measurements for outcome prediction in TAVR patients and found that EAT density is a significant predictor of mortality [8]. In that study, EAT was assessed at the aortic valve (AV)-level, where the important delineation of the pericardium is reliably possible in ECG-gated CT scans, which also makes automated analyses potentially more robust compared to volumetric approaches. However, while various publications address single-slice-based assessment of body composition at the lumbar level, pipelines for highly robust automated analyses of EAT are missing so far [12, 13, 24].

To automatically assess EAT from single-slice CT scans, also slice extraction must be automated in addition to the segmentation step. In the past, different DL approaches have been used to automate this task. For automated body composition analyses at the lumbar level, the selection of a specific slice was considered a segmentation task by taking the landmark as the center of mass of an ellipsoidal segment around the vertebrae [13]. Another approach used a convolutional neural network (CNN) to directly predict the spatial offset of each axial slice to the targeted anatomical landmark [12, 24]. In addition to these classical approaches of supervised learning, promising results have been observed for reinforcement learning (RL)-based methods for the detection of anatomical landmarks [25–27]. RL is the third paradigm of machine learning, alongside supervised and unsupervised learning, and has so far received little attention in image analysis. In RL, an agent learns from interactions with its environment, resulting in a transition to a new state of the environment. Based on the agent’s actions, it receives feedback in the form of positive and negative rewards, which it strives to maximize over time in order to make optimal decisions [28].

In this study, we focus on the development of a single-slice-based assessment of EAT in pre-interventional, ECG-gated spiral CT by automating both required processing steps, namely slice extraction and tissue segmentation. A range of different DL approaches including RL was investigated with the objective of developing a pipeline that enables robust large-scale opportunistic analyses.

## Materials and methods

### Dataset

This retrospective single-center study was approved by the local ethics committee with a waiver for written informed consent. The dataset used for model development consists of patients undergoing TAVR at the University Hospital Bonn with available pre-interventional contrast-enhanced thoraco-abdominal or cardiac CT scans acquired as spiral CT between 2008 and 2020, all prospectively or retrospectively ECG-gated (diastolic phase). Additionally, the trained model was also evaluated on pre-interventional, ECG-gated (diastolic) spiral CT scans of patients receiving CT for pre-operative planning prior to heart surgery. For this evaluation, 50 patients were randomly selected from all corresponding CT scans acquired between July and August 2024.

For the EAT assessment, a single axial slice was selected at the AV-level. Epicardial tissue defined as tissue within the pericardium was manually segmented on this single-slice image and adipose tissue was identified by applying a threshold of −190 to −30 Hounsfield units (HU). Manual data annotation was performed by a radiology resident (B.S.) with 3 years of experience in cardiac imaging supervised by a board-certified radiologist specialized in cardiovascular imaging (D.K.) using the open-source software 3D Slicer (version 4.11) [29].

### Slice extraction

**Fig. 1:**
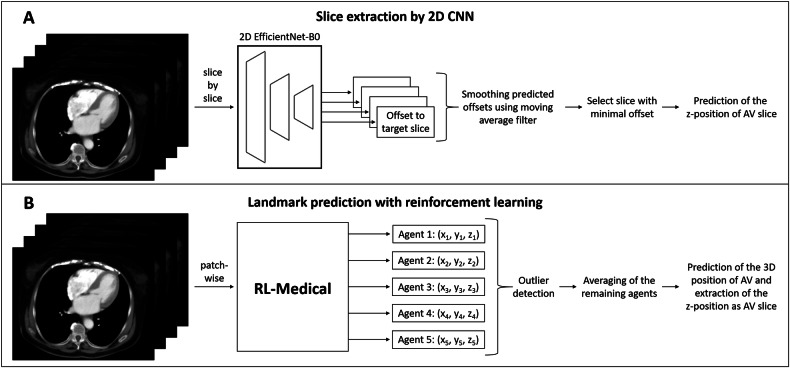
Overview of both slice extraction methods. **A** The baseline approach consists of a 2D convolutional neural network (CNN) using the EfficientNet-B0 architecture and predicts the offset of each axial slice to the target slice at the level of the aortic valve (AV). Predicted offsets were smoothed before the final prediction of the slice with the minimal distance to the target. **B** The reinforcement learning (RL) approach is based on the RL-Medical package. In this case, five agents start at five different random positions, and each agent ends with its own prediction of the 3D AV landmark position. After the application of the outlier detection to the results of the five agents, the final prediction is determined by averaging the landmark predictions of all remaining agents

Two different DL approaches were investigated and compared for automating the slice extraction at the AV-level: A 2D CNN referred to as baseline approach and a 3D RL model. An overview of both methods can be found in Fig. 1.

For the baseline approach, two CNNs following the EfficientNet-B0 architecture were trained to predict the spatial offset of each axial slice to the target AV-level [30]. For the first CNN, the entire dataset was rescaled to a uniform slice thickness of 10 mm to obtain an initial estimate of the actual AV position. Using these rough predictions, the dataset was cropped to the heart region and the second CNN was then trained on high-resolution heart crops to determine the exact slice position. Both CNNs were trained for 1000 epochs using L1-Loss function and AdamW optimizer with a weight decay of 0.01. The learning rate was scheduled by the OneCycle learning rate policy with a maximal learning rate of 10^−^^4^. Training was stopped after no improvement on the validation set was observed for 100 epochs. The performance of the automatic slice extraction was evaluated based on the mean absolute deviation in z-direction (Δz). In a post-processing step, the output predictions of both DL models were smoothed using a moving average filter, where the use of different window sizes was evaluated. For more information on the baseline approach including pre-processing of the data and detailed training parameters, see Supplement S1.

The investigated 3D RL model is based on the open-source software RL-Medical, developed by Alansary et al for 3D landmark detection in medical images [26, 31]. It uses RL in a multi-agent setting, where each agent is positioned within a patch of the 3D medical image considered to be the agent’s state. Agents start exploration from random locations within the image volume, aiming to locate the target through gradual patch movements. The action space, which defines the freedom of motion, allows movement in six directions in 3D Cartesian coordinates. Rewards are based on Euclidean distances (ED) to incentivize movement towards the targeted landmark position. During training, a Q-network is learned as an approximation to the Q-function predicting the next direction of movement by estimating the expected benefit for each action in a state with the goal of attaining the highest possible cumulative reward. The trained network architecture (Network3D) consists of shared convolutional layers among all agents and agent-specific fully connected layers.

Results for the landmark positions were obtained by training the Network3D model with five agents simultaneously searching for the landmark AV. The final AV position was determined by averaging the predicted agents’ end positions, with outlier detection performed on the agents’ positions before averaging. This process involved using z-scores with median absolute deviation to identify and remove outliers [32]. Performance was assessed by calculating the mean ED between the resulting prediction and the target landmark. Additionally, for comparison with respect to the baseline approach, Δz was computed.

More information about the training and details about the utilized outlier detection are described in Supplement S2.

### Tissue segmentation

For automatic segmentation of EAT, a 2D U-Net model with residual units was trained on single-slice images on AV-level using the open-source MONAI python framework (version 0.9.0) [33, 34]. Training was performed for 10,000 epochs using the AdamW optimizer and sum of cross-entropy and Dice coefficient as loss function. A grid search for selection of hyperparameters was conducted to find suitable settings for learning rate (10^−^^3^, 10^−^^4^) and weight decay (10^−^^3^ to 0.5), where the learning rate was adapted for each iteration step according to the OneCycle learning rate policy. A fixed batch size of 200 was used with gradient accumulation over two batches. Training was stopped after no improvement in the validation loss was observed for 100 epochs. The performance of the different hyperparameter settings was compared on the validation set according to the Dice score on which the best model was selected and finally applied to the hold-out test set.

Additionally, the publicly available BOA pipeline was applied to the hold-out test set [19]. The resulting EAT segmentation was considered on the manually selected AV-level only and was compared with ground truth segmentations generated by the radiologist. For more information on the tissue segmentation,  see Supplement S3.

### End-to-end evaluation

Finally, an end-to-end evaluation of the entire pipeline was performed by analyzing and comparing mean absolute difference (MAD) of EAT density (HU) and area (cm^2^) once from automated EAT segmentation at the predicted AV-level and once from manual EAT segmentation at the manually selected AV-level. Therefore, the pipeline was applied to the hold-out test set consisting of spiral CTs from patients undergoing TAVR and to the 50 randomly selected spiral CT scans from patients prior to heart surgery.

### Statistical evaluation

For all performance metrics, 95% confidence intervals (CI) were determined by bootstrapping the test set with 1000 resamples. For end-to-end evaluation, Pearson correlation coefficient was calculated and a Bland-Altman analysis was performed to check for any systematic differences using the Python packages seaborn (version 0.11.2), scipy (version 1.9.1), and pyCompare (version 1.5.3).

## Results

### Dataset

Overall, pre-interventional CT scans from 1583 patients were available, whereby 81 scans were excluded due to artifacts (e.g., caused by metal implants). Thus, 1502 TAVR patients (mean age 80.6 ± 6.3 years, 709 (47.2%) female) were included for method development. Scans were performed on four different scanners, one dual-source CT (Siemens Somatom Force) and three single-source CTs (Philips Brilliance 64, Philips iCT 256, Philips Mx8000 IDT 16). The dataset comprises 948 contrast-enhanced thoraco-abdominal and 554 cardiac computed tomography scans. For method development, the dataset was randomly split into training, validation, and hold-out test cases (*n* = 1052 / 224 / 226). A subset of the data has already been used in another study investigating the correlation between inflammatory changes in EAT or PAT and cardiac risk [8]. The 50 randomly selected pre-interventional CT scans from patients undergoing heart surgery (mean age 63.9 ± 15.7 years, 18 (36.0%) female) were used as additional test set for the end-to-end evaluation. The dataset comprises 25 cardiac CT scans performed on Siemens Somatom Force (Test A) and 25 contrast-enhanced thoraco-abdominal scans performed on Siemens Naeotom Alpha (Test B). For information on image characteristics, see Table 1.

**Table 1:** Detailed overview of image characteristics of the dataset, including pixel spacing, slice thickness, matrix size, and tube voltage

	TAVR data		Test A		Test B	
	Median	Range	Median	Range	Median	Range
Pixel spacing (mm)	0.46	[0.27, 0.98]	0.39	[0.34, 0.49]	0.62	[0.58, 0.64]
Slice thickness (mm)	1	[0.6, 3]	1	[1, 1]	1	[1, 1]
Matrix size	512	[512, 768]	512	[512, 512]	576	[512, 577]
Tube voltage (kV)	120	[70, 140]	90	[80, 120]	140	[140, 140]

### Slice extraction

For the baseline approach, a moving average filter with a window size of 11 led to the highest performance on the validation set with Δz = 2.1 ± 1.9 mm. More details on applying the moving average filter using different window sizes can be found in Supplement S4. The application to the hold-out test set resulted in a mean deviation of 2.0 ± 2.0 mm (95% CI: [1.8, 2.3] mm).

The RL approach resulted in a mean ED of 3.4 ± 4.2 mm (max ED = 59.5 mm) and a mean Δz of 1.9 ± 3.8 mm (max Δz = 54.0 mm). The application of the outlier detection method before averaging the positions of the five agents improved the performance regarding both metrics (mean ED distance = 3.3 ± 1.9 mm, max ED = 10.0 mm; mean Δz = 1.7 ± 1.5 mm, max Δz = 7.0 mm). Applying this approach to the hold-out test set led to even smaller Δz = 1.8 ± 1.6 mm (95% CI: [1.6, 2.0] mm) compared to the baseline approach. Mean ED on the hold-out test set was 3.3 ± 2.2 mm (95% CI: [3.0, 3.6] mm). Table 2 provides a detailed comparison of both methods.

**Table 2:** Performance overview for both slice extraction approaches on the hold-out test set

	Baseline approach	RL-Medical	
	Δz	Δz	ED
Mean ± std	2.0 ± 2.0	1.8 ± 1.6	3.3 ± 2.2
Min	0	0	0
Max	12.2	7	14.6
10th percentile	0	0	1.4
90th percentile	4.0	4.0	5.5

All metrics are provided in mm. For the baseline approach, absolute deviation in z-direction (Δz) is given, and for the reinforcement learning approach (RL-Medical) the Euclidean distance (ED) was additionally determined

### Tissue segmentation

Highest performance for the implemented 2D U-Net on the validation set was reached after 1512 epochs with a maximal learning rate of 10^−^^3^ and a weight decay of 0.1 (see Supplement S5). Mean Dice score was 91.6 ± 4.2%. Applying the model to the hold-out test set yielded a Dice score of 91.3 ± 4.4% (95% CI: [90.7, 91.8] %). The agreement at AV-level of the open-source pipeline BOA with the radiologist’s segmentation showed a significantly lower Dice score of 85.6 ± 7.1% (95% CI: [84.7, 86.5] %). The 2D U-Net also showed significantly higher agreement with the radiologist regarding MAD of density and area than the BOA pipeline (see Table 3).

**Table 3:** Comparison of the implemented 2D U-Net and the open-source body and organ analysis (BOA) pipeline with mean ± standard deviation and 95% confidence intervals in the brackets

	2D U-Net vs. GT	BOA vs. GT
Mean Dice score (%)	91.3 ± 4.4 [90.7, 91.8]	85.6 ± 7.1 [84.7, 86.5]
Mean absolute density difference (HU)	1.6 ± 1.4 [1.4, 1.7]	3.7 ± 3.4 [3.3, 4.1]
Mean absolute area difference (cm^2^)	1.0 ± 1.0 [0.8, 1.1]	1.9 ± 1.7 [1.7, 2.1]

The comparison contains the mean Dice score in %, the mean absolute density difference in Hounsfield units (HU), and the mean absolute area difference in cm^2^. Note that this is a comparison at a single axial slice, where ground truth (GT) was defined at the level of the aortic valve

### End-to-end evaluation

**Fig. 2:**
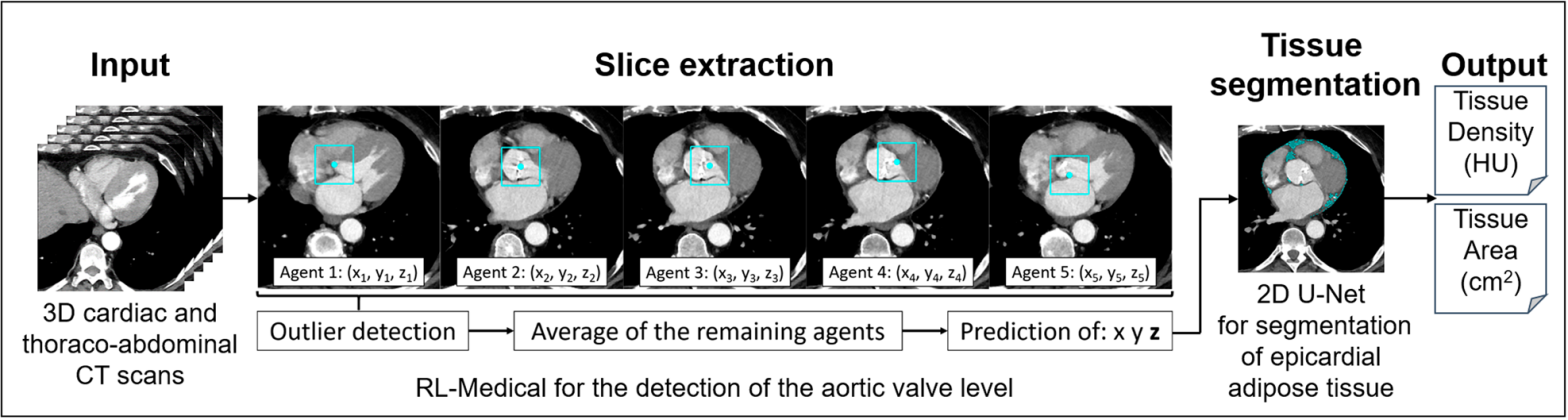
Overview of the final pipeline consisting of the reinforcement learning (RL) method for slice extraction of the aortic valve level and a 2D U-Net for segmentation of the epicardial adipose tissue

Based on the validation results, a pipeline comprising RL-based slice extraction with outlier detection and 2D U-Net-based tissue segmentation was chosen for end-to-end evaluation. An overview of the final pipeline can be found in Fig. 2. The training and inference script are accessible at https://github.com/ukb-rad-cfqiai/EAT_Assessment.

**Fig. 3:**
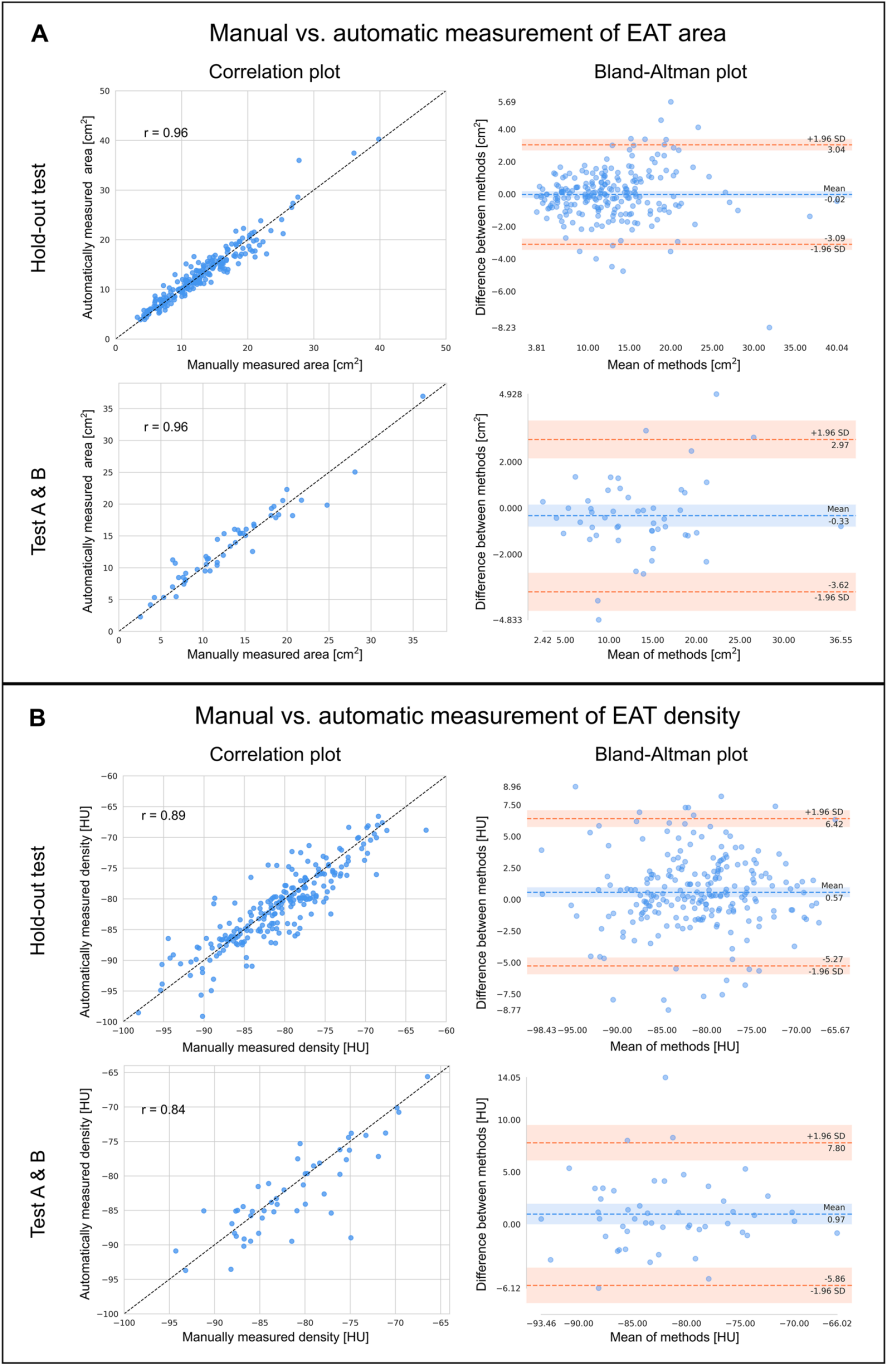
Correlation and Bland-Altman analyses for the end-to-end comparison of manually and automatically measured epicardial adipose tissue (EAT) area given in cm^2^ (**A**) and density given in Hounsfield units (HU) (**B**) for the hold-out test set consisting of pre-interventional TAVR patients and the test sets (Test A and Test B) consisting of pre-operative patients prior to heart surgery. The Pearson correlation coefficient (*r*) is provided for all analyses

A high agreement was observed between automated and manual assessment of EAT at the AV-level for the hold-out test set (MAD density = 2.2 ± 2.1 HU (95% CI: [2.0, 2.5] HU), MAD area = 1.1 ± 1.1 cm^2^ (95% CI: [1.0, 1.3] cm^2^)). A significant correlation was found between automatically and manually measured EAT area with a Pearson correlation coefficient of *r* = 0.96 (*p* < 0.001) and between automatically and manually measured EAT density (*r* = 0.89, *p* < 0.001). Bland-Altman analysis revealed no systematic deviation between manual and automatic end-to-end comparison, with low mean area difference of −0.02 cm^2^ and low mean density difference of 0.57 HU (see Fig. 3). Similar performance was observed for the additional test sets Test A and Test B consisting of pre-operative CT scans (see Table 4 and Fig. 3).

**Table 4:** End-to-end comparison of the final pipeline evaluated on the hold-out test set containing pre-interventional TAVR patients, the pre-operative cardiac (Test A) and the pre-operative thoraco-abdominal (Test B) scans

	Hold-out test set	Test A	Test B
Mean absolute density difference (HU)	2.2 ± 2.1 [2.0, 2.5]	2.0 ± 1.8 [1.4, 2.7]	2.8 ± 3.0 [1.9, 4.1]
Mean absolute area difference (cm^2^)	1.1 ± 1.1 [1.0, 1.3]	1.0 ± 0.9 [0.7, 1.4]	1.4 ± 1.3 [1.0, 1.9]

The performance is given as mean ± standard deviation and 95% confidence intervals for the mean absolute density difference in Hounsfield units (HU) and the mean absolute area difference in cm^2^

**Fig. 4:**
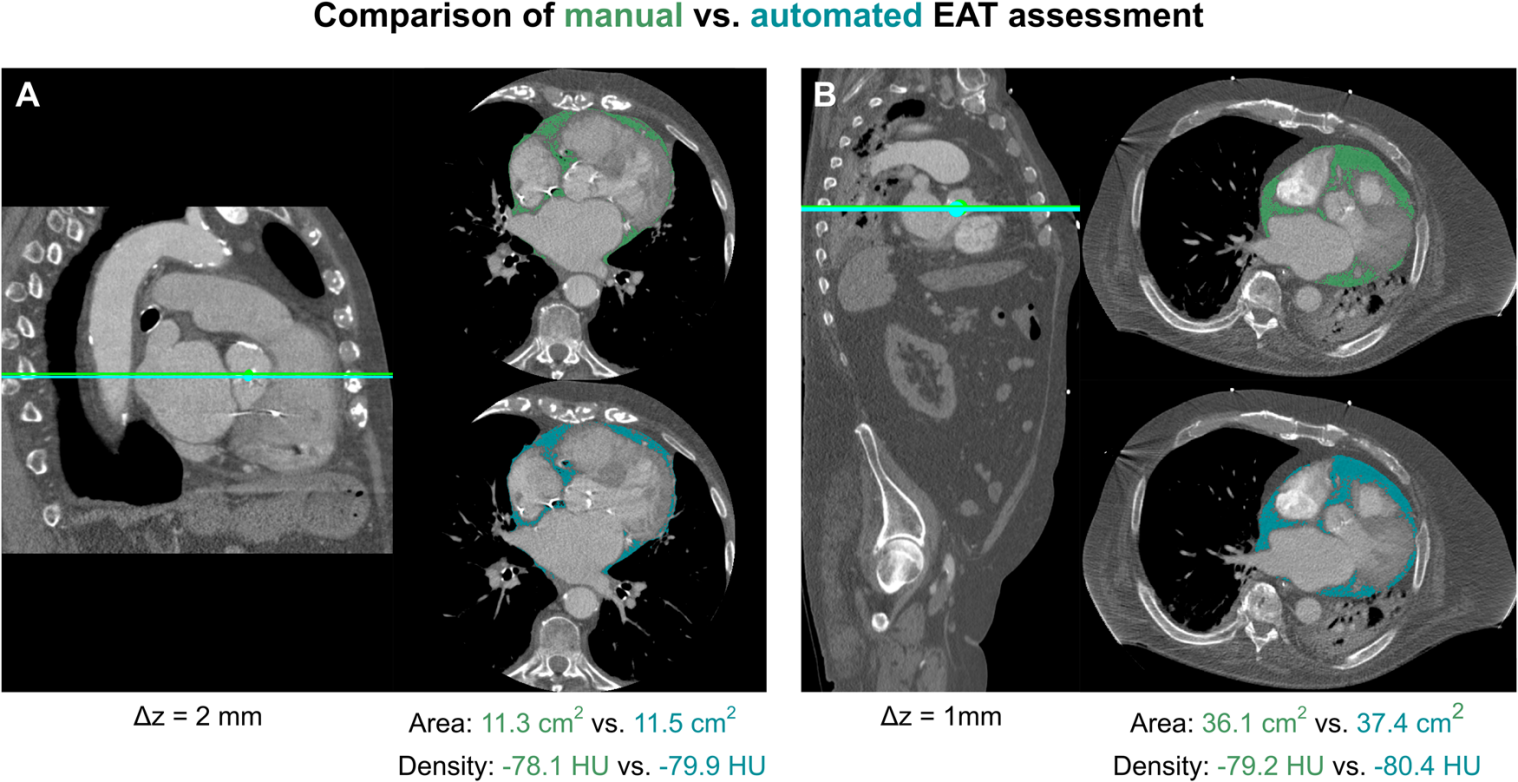
Two examples of the end-to-end comparison of manual (marked in green) and automated (marked in blue) epicardial adipose tissue (EAT) assessment for a contrast-enhanced cardiac CT (**A**) and an ECG-gated thoraco-abdominal CT scan (**B**). Δz denotes the absolute difference between manually selected and automatically predicted slice position at the level of the aortic valve

Figure 4 presents examples of the end-to-end evaluation in a cardiac and a thoraco-abdominal scan. Manual segmentation time using the open-source software 3D Slicer took about 100 s per patient. In comparison, the inference time for RL-based slice extraction was about 30 s per patient for cardiac CTs and 127 s for thoraco-abdominal CT scans. Time for automatic segmentation was only 2 s per patient. Inference time was measured on an Nvidia GeForce RTX 3090 Graphics Processing Unit with 24 gigabyte video memory.

## Discussion

This paper presents an automated pipeline for opportunistic assessment of EAT from contrast-enhanced ECG-gated (diastolic) cardiac and thoraco-abdominal spiral CT scans for pre-interventional patients undergoing TAVR or heart surgery. The proposed approach essentially consists of two steps: automatic slice extraction at the AV-level and 2D segmentation of EAT.

For the slice extraction task, the RL approach achieved impressive accuracy. In contrast to the baseline approach, which aims to predict the z-distance of a slice to the targeted slice, the RL model utilizes 3D data, likely explaining its advantages over the 2D approach, which receives only a single axial slice as input. In addition, a multi-agent setting was used for the RL approach, i.e., the position of the landmark is predicted by five agents during inference. The ensembling-like averaging of the five agents after outlier detection contributed to a more robust and generalizable model. For tissue segmentation at AV-level, the 2D U-Net model achieved a higher precision for EAT segmentation compared to the volumetric segmentation approach, resulting in high agreement between manually and automatically measured area and mean density in the end-to-end evaluation.

Various approaches to automated EAT assessment have been explored in the past, ranging from traditional methods to DL algorithms to avoid time-consuming and tedious manual tasks. One approach for this purpose is atlas-based segmentation, in which the input data is registered to a set of reference images [35–37]. In recent years, DL methods have become established for segmentation tasks and have also already been implemented for EAT assessment. In 2019, Commandeur et al presented a multi-task DL framework for EAT segmentation in non-contrast-enhanced cardiac CTs, which takes three consecutive axial slices as input. In the first task, the DL algorithm classifies whether the central input slice is located in the heart region while in the second, the pericardium is segmented [3, 4, 16, 38]. Hoori et al proposed DeepFat, a CNN with atrous convolution for automatic segmentation of EAT in CT calcium score images. Further approaches used either generative adversarial networks or various U-Net-based architectures for segmentation [17, 18, 39–41]. Recently, the open-source DL model called BOA was introduced which extends classical body composition analysis to volumetric assessment of cardiac adipose tissue in CT scans [19].

In our work, we focused on automated 2D EAT assessment at the AV-level in pre-interventional CT scans of patients undergoing TAVR, as a correlation between mean EAT density measured at this single-slice and overall survival has recently been reported in this cohort [8]. In addition, we have shown that our algorithm also enables robust EAT evaluation on pre-operative CT data prior to heart surgery. A major difficulty in segmenting the entire EAT is the differentiation between pericardial and epicardial tissue, as the pericardium cannot always be clearly delineated on every CT image. Thus, significant differences have been found in the past when comparing the EAT assessment of two readers, and a high inter-reader variability of 15% has been reported for EAT volume measurement [16, 42]. In contrast, a low inter-reader variability of 0.1% was reported at the AV-level for both the measured EAT area and the mean density, suggesting that the delineation of epicardial tissue on this slice is more robust and reliable, which is very likely also true for automated opportunistic assessment [8]. This aspect probably explains why the proposed 2D approach was superior to the 3D BOA pipeline, as our method was explicitly trained at AV-level, where EAT segmentation can be performed with higher reliability. However, it remains an open question as to which method is most appropriate in terms of developing predictive models. In particular, EAT volume has been shown in many studies to be a prognostic marker for overall survival in various cardiac diseases, and it remains unclear whether the robust determination of EAT area as a surrogate for EAT volume or the potentially less robust direct determination of EAT volume is better suited for the development of predictive models [1–7]. It is therefore a future task to investigate the impact of 2D versus 3D measurements for outcome analysis.

Our study has several limitations. First, all investigated methods were developed on images from diastolic phases acquired in ECG-gated spiral technique. Future studies should address whether the presented algorithm is also applicable to coronary CT angiography (CCTA) data. In contrast to pre-interventional CT for planning of TAVR and surgery, CCTA data are typically acquired with step-and-shoot technique and reconstructed in various heart phases. Images acquired in step-and-shoot frequently show step artifacts, potentially affecting automated localization. This may require the inclusion of such data in the training set. Moreover, data was obtained from only one center. Although methods were developed based on various CT scans with different scan lengths (ECG-gated thoraco-abdominal and cardiac CT scans), a multi-center study is desirable to further verify the generalizability. Even though this study considered a heterogeneous dataset, it did not examine how scanning protocol differences and image quality affect the EAT measurement. In addition, only contrast-enhanced CT scans were included in our study, as these are part of the pre-interventional diagnostic work-up in patients undergoing TAVR or heart surgery.

In conclusion, we propose a pipeline for robust automatic 2D assessment of EAT in pre-interventional, ECG-gated spiral CT scans. End-to-end automation eliminates the need for any manual interaction and enables an opportunistic large-scale assessment of EAT.

## Supplementary information

ELECTRONIC SUPPLEMENTARY MATERIAL

## Data Availability

The trained models can only be shared for research purposes on request due to the German Data Protection Law.

## Abbreviations

AV: Aortic valve
BOA: Body and organ analysis
CAT: Cardiac adipose tissue
CI: Confidence intervals
CNN: Convolutional neural network
CCTA: Coronary computed tomography angiography
DL: Deep learning
EAT: Epicardial adipose tissue
ED: Euclidean distance
HU: Hounsfield units
MAD: Mean absolute difference
PAT: Pericardial adipose tissue
RL: Reinforcement learning
TAVR: Transcatheter aortic valve replacement

## Code availability

The code used for this study is publicly available at https://github.com/ukb-rad-cfqiai/EAT_Assessment, which allows for reproducing the described experiments with own data.
