# Supplementary material for: Deep learning for opportunistic, end-to-end automated assessment of epicardial adipose tissue in pre-interventional, ECG-gated spiral computed tomography
Source: Insights Imaging. 2024 Dec 19;15:301. doi: 10.1186/s13244-024-01875-6 (PMC11659545; doi:10.1186/s13244-024-01875-6)
Supplement: Supplementary file 1 — ELECTRONIC SUPPLEMENTARY MATERIAL [file 13244_2024_1875_MOESM1_ESM.pdf]

Deep learning for opportunistic, end-to-end automated assessment of epicardial adipose tissue in pre-interventional, ECG-gated spiral computed tomography

ELECTRONIC SUPPLEMENTARY MATERIAL

Supplement S1 Experimental Design: Slice Extraction – Baseline Approach

For the first convolutional neural network (CNN) of the baseline approach, all CT slices were first resized to a matrix size of 256x256 using cubic interpolation with a uniform slice thickness of 10mm, where the landmark position of the aortic valve (AV) coordinate was transformed accordingly. For the second CNN, the predicted AV position of the previous deep learning (DL) model was used to crop CT scans to the heart region in the sagittal plane using the predicted position ±10cm as the upper and lower limit. Boundaries of the heart crop in the axial and coronal plane were identified using the center of mass ± 15cm within the body mask determined by morphological operations. Finally, all CT slices were reshaped to a uniform slice thickness of 1mm, resulting in a matrix size of 300x300.

For both CNNs, morphological opening was performed with a 3x3 kernel with a subsequent connected component algorithm to select only the largest segment. Additionally, the Hounsfield Units (HU) were clipped to a minimum value of -400 HU and a maximum value of 600 HU and finally, min-max normalization was applied to transform all intensity values into a range between zero and one.

For training, the offset of the current input slice position to the AV slice was used as ground truth, which was transformed into a range of [0, 1] using a stretched sigmoid function with $\sigma = 25$ for the first CNN and $\sigma = 10$ for the second EfficientNet-B0 [1]:

$$r(z) = \frac{1}{1 + e^{-\sigma(z - z_{AV})}}$$

In this way, the model learns to predict the target $r(z)$, where z refers to the slice number of the current axial input slice and z_{AV} to the slice position of the manually labeled AV coordinate.

The first CNN was trained with a batch size of 128 with gradient accumulation over 10 batches. Training was performed in PyTorch (version 1.12.1, Cuda 11.3) with mixed precision on an Nvidia GeForce RTX 3090 Graphics Processing Unit (GPU) with 24 gigabyte (GB) video memory. For the second CNN trained on high-resolution heart crops, a batch size of 400 with gradient accumulation over 3 batches was used. Here, training was executed in PyTorch (version 2.1.2, Cuda 12.1) with mixed precision on an Nvidia A100 GPU with 80 GB video memory.

Supplement S2 Experimental Design: Slice Extraction – Reinforcement Learning Approach

<u>Part 1 – Landmark detection</u>

The reinforcement learning (RL) approach utilised in this study is the 'RL-Medical' software from the open-access GitHub repository (https://github.com/gml16/rl-medical) [2]. For this method, the input data was reshaped to a homogenous voxel size of 1x1x1mm^3 and the ground truth 3D target position of the AV landmark was rescaled accordingly.

For this work, the Network3D architecture from the RL-Medical framework was chosen. It utilizes a siamese neural network with four convolutional and three pooling layers, which are shared among all five agents. The input was transformed into a range of [0, 1] and has the size *batch_size x number_agents x patch_size x history_length*. For the training phase of 24 epochs, a batch size of 100 and 5 agents were employed. The patch size has a shape of 45x45x45, and the history length is 4 defining the individual state of the agents.

The results of this CNN are forwarded to the classification part consisting of five independent fully connected layers, which equals number of agents. Each layer supplies the corresponding agent with the Q-vector comprising six elements for the possible freedom of movement.

The patch is defined as the region of interest of the agent. In the beginning, the patch had a physical extent of 135x135x135 mm^3, which was gradually reduced to 90x90x90 mm^3 and then to 45x45x45 mm^3 after each oscillation. The terminal state was achieved when in the final physical state the maximum number of allowed oscillations was reached, which was set to 8 within the last 20 steps, or the Euclidian distance (ED) between all agents' positions and AV was equal or less than 1mm for every agent.

In each episode, 300 steps per agent were possible. The training frequency was set to every 10 steps of the agent and the network was saved every 10 training steps. The experience replay buffer had a capacity of 10^5, with an initial memory size of $3*10^4$. The best location with the best q-value was obtained from the last 10 locations stored in the history.

The epsilon-greedy strategy was used for training with an epsilon value of 1, which was reduced by 10^{-4} after each episode until the minimum of 0.1 was reached. The mean value of all agents was added to the rewards. The discount factor in the Bellman equation was set to 0.9, with a learning rate of 10^{-4}. After every 10 epochs, the learning rate was reduced by a factor of 0.05.

The experiments were performed using PyTorch (version 3.9.16, Cuda 12.2) on an Nvidia GeForce RTX 3090 GPU with 24 GB of video memory.

Part 2 – Outlier detection

The RL-model predicts a 3D landmark position targeting the landmark AV for every agent separately. This provides five predictions that can be used to reduce the influence of statistical fluctuations on the final landmark prediction. An outlier detection was used to prevent influence of possible outliers in the agents' positions which can be caused by suboptimal starting positions of an agent or individual patients' conditions (e.g. implants along the path of the agent).

Since only 5 data points were available for the prediction, the 'median absolute deviation' (MAD) approach was used to detect outliers, which is a simple but robust solution that works with few data points [3, 4]. MAD is defined as the median absolute deviation about the median (med_i) with $med_j(x_j)$ being the median of the dataset x_j of n observations and b as a multiplicator.

$$MAD_n = b \cdot med_i \, |x_i - med_j(x_j)|$$

The constant parameter b is specified as $b = 1/Q(0.75)$ with $Q(0.75)$ as the 0.75 quantile of the underlying distribution. After analysing the data distribution of the evaluation data set, it was determined that the data follows an exponential distribution, see Figures S1 and S2. Therefore, b was set to 0.7213 with $Q(0.75)$ of the exponential distribution.

To check for outliers, the following calculation for each x_i was determined and those x_i exceeding a certain threshold (here set to ±2.5) were marked as outliers:

$$zScore = \frac{\left|x_i - med_j(x_j)\right|}{MAD_n}$$

This z-score filtering was performed for each dimension and as soon as an outlier was detected in one dimension, the entire point was labeled as an outlier.

To address cases where data points might have been incorrectly classified as outliers due to the robust nature of MAD, an additional check ensures that only data points with a Euclidean distance >15mm from the mean of the other data points are labeled as true outliers.

In general, the outlier detection algorithm was only applied when the absolute difference between the maximum and minimum data point in any dimension was >15 mm.

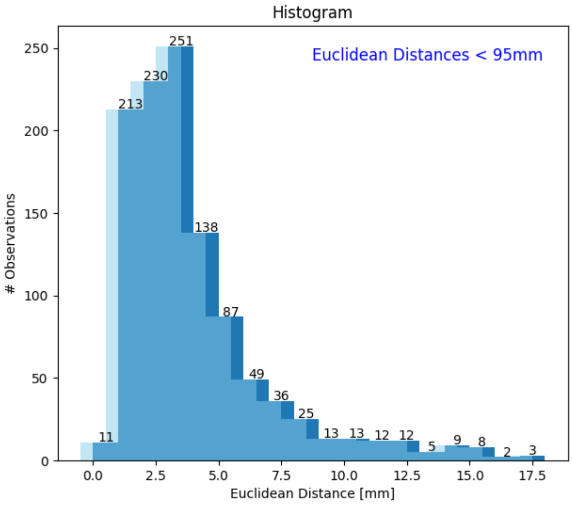

Figure S1: Histogram of the Euclidean distances [0:95mm] between all agents separately and the target landmark aortic valve.

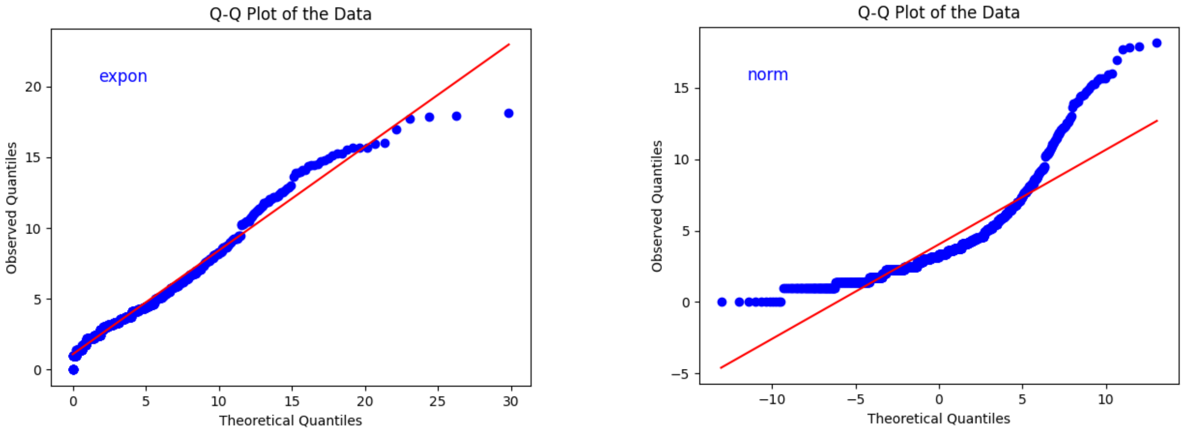

Figure S2: Q-Q Plot of the Euclidean Distances [0:95mm] between all agents separately and the target landmark aortic valve. Left: Data as exponential distribution. Right: Data as normal distribution.

Supplement S3 Experimental Design: Tissue Segmentation

For tissue segmentation, CT slices were pre-processed by reshaping to a fixed matrix size of 512x512 and clipping all HUs into a range of [-400; 600] HU. Body mask was identified using *findContours* function from OpenCV (version 4.5.5) together with morphological operations. A min-max normalization was applied to transform all intensity values into a range between zero and one. For training the tissue segmentation, all experiments were performed in PyTorch (version 2.1.2, Cuda 12.1) with mixed precision on an Nvidia A100 GPU with 80 GB video memory.

Supplement S4 Results slice extraction: Baseline approach

For both CNNs in the baseline approach, the use of a moving average filter was investigated as a post-processing step to smooth the networks' outcome predictions. In Table S4, the results for both CNNs are listed after applying a moving average filter with different window sizes to the validation set.

Model	Moving Average Window Size	Mean Δz [mm]	Min Δz [mm]	Max Δz [mm]
Rough Slice Extraction	-	5.8 ± 7.4	0	80
	3	3.3 ± 7.0	0	80
	**5**	**2.8 ± 4.6**	**0**	**20**
	7	3.0 ± 4.7	0	20
	9	3.0 ± 4.7	0	20
	11	3.0 ± 4.7	0	20
Exact Slice Extraction	-	2.3 ± 3.7	0	49
	3	2.3 ± 3.7	0	49
	5	2.3 ± 3.7	0	49
	7	2.3 ± 3.7	0	49
	9	2.3 ± 3.6	0	49
	**11**	**2.1 ± 1.9**	**0**	**9**

Table S4 Results on the validation set after applying a moving average filter to the predictions from the rough slice extracting CNN trained to crop CT scans to the heart and the exact slice predicting CNN trained on high-resolution heart crops. The table lists the mean deviation in z-direction (Δz), together with minimal and maximal observed deviation. The highest performance for each model is marked in bold.

Supplement S5 Grid search for tissue segmentation

Learning Rate	Weight Decay	Min Valid Loss	Epoch	Dice
0.0001	0.001	0.1610	4227	90.17%
	0.005	0.1599	4145	90.38%
	0.01	0.1607	4418	90.23%
	0.05	0.1599	4219	90.54%
	0.1	0.1609	4406	90.29%
	0.5	0.1626	4664	90.38%
**0.001**	0.001	0.1873	1190	89.95%
	0.005	0.1507	1478	90.65%
	0.01	0.1754	1246	90.06%
	0.05	0.1495	1514	90.63%
	**0.1**	**0.1527**	**1512**	**91.56%**
	0.5	0.1839	1285	91.08%

Table S5 Performance overview on the validation set with different hyperparameter settings. Highest performance was observed using a leaning rate of 10^{-3} and a weight decay of 0.1 after 1512 epochs (marked in bold).

References

1. Bridge CP, Rosenthal M, Wright B, et al (2018) Fully-Automated Analysis of Body Composition from CT in Cancer Patients Using Convolutional Neural Networks. In: Stoyanov D, Taylor Z, Sarikaya D, et al (eds) OR 2.0 Context-Aware Operating Theaters, Computer Assisted Robotic Endoscopy, Clinical Image-Based Procedures, and Skin Image Analysis. Springer International Publishing, Cham, pp 204–213

2. Leroy G, Rueckert D, Alansary A (2020) Communicative Reinforcement Learning Agents for Landmark Detection in Brain Images. In: Kia SM, Mohy-ud-Din H, Abdulkadir A, et al (eds) Machine Learning in Clinical Neuroimaging and Radiogenomics in Neuro-oncology. Springer International Publishing, Cham, pp 177–186

3. Rousseeuw PJ, Croux C (1993) Alternatives to the Median Absolute Deviation. Journal of the American Statistical Association 88:1273–1283. https://doi.org/10.1080/01621459.1993.10476408

4. Leys C, Ley C, Klein O, et al (2013) Detecting outliers: Do not use standard deviation around the mean, use absolute deviation around the median. Journal of Experimental Social Psychology 49:764–766. https://doi.org/10.1016/j.jesp.2013.03.013
